# Complete Genome Sequence of Sterol-Transforming *Mycobacterium neoaurum* Strain VKM Ac-1815D

**DOI:** 10.1128/genomeA.01177-13

**Published:** 2014-01-16

**Authors:** Victoriya Y. Shtratnikova, Eugeny Y. Bragin, Dmitry V. Dovbnya, Yury A. Pekov, Mikhail I. Schelkunov, Nicolai Strizhov, Tanya V. Ivashina, Vasily V. Ashapkin, Marina V. Donova

**Affiliations:** Center of Innovations and Technologies Biologically Active Compounds and Their Applications, Moscow, Russiaa; Institute of Biochemistry and Physiology of Microorganisms, Russian Academy of Sciences, Pushchino, Russiab; Department of Bioengineering and Bioinformatics, Lomonosov Moscow State University, Moscow, Russiac; Belozersky Institute of Physico-Chemical Biology, Lomonosov Moscow State University, Moscow, Russiad

## Abstract

*Mycobacterium neoaurum* strain VKM Ac-1815D produces 4-androstene-3,17-dione as a major compound from phytosterols. Here, we report the complete genome sequence of the strain. The genome consists of a single circular 5,438,190-bp chromosome, with a G+C content of 66.88%, containing 5,318 putative open reading frames (ORFs), 46 tRNAs, and 6 rRNAs. Arrays of cholesterol metabolism genes are randomly clustered throughout the chromosome.

## GENOME ANNOUNCEMENT

A number of mycobacterial strains with an origin in soil effectively oxidize cholesterol and phytosterols (1, 2). *Mycobacterium* sp. strain VKM Ac-1815D is of particular interest because it is capable of synthesizing the valuable steroid precursor 4-androstene-3,17-dione as a major product from sitosterol (3). Based on phylogenetic analysis of 16S rRNA sequences (4) and chemotaxonomy, the strain was reclassified as *Mycobacterium neoaurum* VKM Ac-1815D.

Two genomic DNA fragment libraries of the strain were generated and read on a Genome Analyzer IIx (paired-end 72-nucleotide reads) and on HiSeq 2000 (paired-end 100-nucleotide reads) as per the protocols of the manufacturer (Illumina), with 500 to 800× coverage. The short-read library contained DNA fragments of 400-bp mean length prepared with a DNA sample preparation kit (New England BioLabs) after digestion by NEBNext dsDNA Fragmentase. The mate-pair library with 2,000- to 10,000-bp-long fragments was created with the techniques recommended by the supplier of the Nextera mate-pair sample preparation kit (Illumina).

The short reads and mate-pair reads obtained in a FASTQ Illumina 1.5+ format were used for the *de novo* genome assembly with Velvet 1.2 (5) and SPAdes 2.5 (6). Contig sequences up to 575 kbp in length were produced (30 to 40 contigs depending on the assembler used). The circularization of the genome, gaps in scaffolds, and ambiguities were resolved by primer walking to the edges of the contigs, followed by Sanger sequencing of the PCR-amplified DNA fragments. All generated reads were assembled in a single sequence representing the complete bacterial chromosome. Genome assembly validation was performed by mapping of the reads with the Burrows-Wheeler Aligner (BWA) program (http://bio-bwa.sourceforge.net/).

The genome consists of a single circular 5,438,190-bp chromosome with a G+C content of 66.88% and encodes 46 tRNAs and 6 rRNAs (5S, 23S, and 16S in two copies each located in two rRNA loci). Annotation of the genome by the Internet service xBASE (http://www.xbase.ac.uk/annotation) revealed 5,318 open reading frames (ORFs) with *Mycobacterium tuberculosis* H37Rv used as a reference strain and 5,192 ORFs with *Mycobacterium smegmatis* mc^2^ 155 as a reference strain; i.e., the numbers of putative ORFs in the complete genome are greater than those found in the draft sequence (4). A preliminary analysis of the genome sequence shows that arrays of genes involved in cholesterol metabolism (side chain degradation, central pathway, and transport) are distributed throughout the bacterial chromosome, randomly located in different clusters.

The reported genome sequence will contribute not only to the elucidation of cholesterol metabolism in actinobacteria but also to the development of new biotechnological applications for mycobacterial conversion of important bioactive steroids.

### Nucleotide sequence accession number.

The complete genome sequence has been deposited in GenBank under the accession no. CP006936.
